# Synergistic effect of rice bran extract and extremely low‐frequency electromagnetic fields on dermal papilla/melanocytes in melanogenesis

**DOI:** 10.1002/bem.22151

**Published:** 2018-10-29

**Authors:** Soon‐Joung Kwon, Yu‐Mi Kim, Hyun‐Jun Jang, Young‐Kwon Seo

**Affiliations:** ^1^ Department of Medical Biotechnology (BK21 Plus team) Dongguk University Gyeonggi‐Do Korea

**Keywords:** dermal papilla, electric magnetic field, melanocyte, melanogenesis, rice bran extract, spheroid culture

## Abstract

Melanocytes in hair are located around dermal papilla cells at the tip of the hair follicle. In this study, we examined the melanogenesis of a three‐dimensional (3D) hair dermal papilla model treated with natural extracts and electromagnetic fields (EMFs). The 3D model involved dermal papilla‐like tissue (DPLT), an aggregation of a mixture of dermal papilla cells, and melanocytes in microwells. Rice bran extract (RBE), an EMF, and RBE/EMF were applied to different DPLT groups. The LDH assay indicated no cell stress in all experimental groups, and detection of tyrosinase activity demonstrated high activity in the RBE/EMF group. Western blot analysis of the RBE, EMF, and RBE/EMF groups revealed increased MITF, TRP‐1, and tyrosinase expression. In addition, the mRNA expression of ET‐1, laminin, bFGF, β‐catenin, MITF, and tyrosinase was increased in the RBE/EMF group, as demonstrated by RT‐qPCR analysis. HMB45 and Fontana–Masson immunostaining showed that the RBE/EMF group had the highest melanin content. Therefore, RBE and EMF may be used as a material and therapy, respectively, for the treatment of vitiligo and white hair, through activation of melanogenesis in melanocytes. Bioelectromagnetics. 39:595–603, 2018. © 2018 The Authors. *Bioelectromagnetics* Published by Wiley Periodicals, Inc..

## INTRODUCTION

In the human scalp, hair follicles display cyclical activity with each cycle being characterized by three successive phases (anagen, catagen, and telogen) that have distinct durations [Commo and Bernard, 2000]. One of the characteristics of the human hair shaft is the presence of melanin in the cortex, which is responsible for hair color. Melanocytes are the only cells capable of synthesizing melanin in mammalian hair and skin; these cells are responsible for hair shaft pigmentation and are located in the bulb of the hair follicle [Commo and Bernard, 2000]. Melanocytes can be found in the basement membrane surrounding the dermal papilla at the apex, and their dendrites extend to pre‐cortical keratinocytes, defining the so‐called hair melanin unit [Commo and Bernard, 2000].

In hair, melanin is a compound that determines hair color. Gray hair is affected not only by genetic factors but also by strong ultraviolet (UV) light and stress. Melanogenesis is a physiological process resulting in the production of melanin, which plays an important role in the prevention of sun‐induced skin injury and contributes to skin and hair color [Cho et al., 2016]. Recently, various natural materials have been found to lead to melanogenesis, such as extracts of *Ardisia crenata*, scoparone, mangosteen leaf, and Kaliziri [Cho et al., 2016]. Jang and Seo [2016] reported that rice bran extract (RBE) is comprised of specific minerals that promote melanin synthesis through microphthalmia‐associated transcription factor (MITF) and cyclic AMP (cAMP)‐responsive element binding protein (CREB) phosphorylation in melanocytes.

It is widely known that extremely low‐frequency electromagnetic fields (ELF‐EMFs) can interact with biological systems and affect health [Cho et al., 2016]. ELF‐EMFs have been suggested to influence several types of cells and processes, including cell migration, differentiation, apoptosis, and stress responses [Cho et al., 2016]. Kim et al. [2017] investigated whether 60 Hz of ELF‐EMFs could stimulate the biosynthesis of melanin by promoting tyrosinase and tyrosinase‐related protein 1 (TRP‐1). In addition, Cho et al. [2016] reported that ELF‐EMFs could increase phosphorylated CREB (p‐CREB), resulting in the enhanced expression of MITF and downstream factors TRP‐1, tyrosinase‐related protein 2 (TRP‐2), and tyrosinase in melanocytes.

Pigment production and deposition are restricted to the hair follicle and hair shaft, respectively. During the active growth phase (anagen) of the mature hair follicle, pigments are synthesized by melanocytes in the hair bulb at the base of the follicle and adjacent to the dermal papilla, a specialized mesenchymal component of the hair follicle that plays important roles in controlling follicle morphogenesis, stem cell activity, hair shaft formation, and pigmentation [Enshell‐Seijffers et al., 2010]. Therefore, in this study, melanocytes and dermal papilla cells were co‐cultured with three‐dimensional (3D) spheres to generate dermal papilla‐like tissue (DPLT) for cell experiments.

We prepared DPLT as 3D aggregates similar to those under in vivo conditions, and a hair model was produced by adhering melanocytes to the DPLT. The effects of RBE and EMFs on melanogenesis were examined, and the synergistic effects of simultaneous stimulation with RBE and EMF were evaluated. After DPLT culture, tyrosinase activity and lactate dehydrogenase (LDH) were analyzed, and western blot analyses of MITF, TRP‐1, tyrosinase, and p‐CREB were performed. Furthermore, the mRNAs of endothelin‐1 (ET‐1), laminin, basic fibroblast growth factor (bFGF), β‐catenin, MITF, and tyrosinase were analyzed, and HMB45 and Fontana–Masson immunostainings were assessed.

## MATERIALS AND METHODS

RBE was produced using the method described by Jang and Seo [2016]. RBE was obtained from the carbonized chaff of rice bran. Rice bran ash (200 g) was added to 1 L of distilled water and stirred at 400 rpm and 100 °C for 24 h. Next, the mixture was filtered through 10 μm filter paper, centrifuged at 10,000 rpm at 25 °C for 30 min to remove any remaining particulate matter, and then sterilized using a 0.2 μm syringe filter.

This study was approved by the Institutional Review Board of Dongguk University (DUIRB‐20151127‐011, 31 December 2017). Human hair dermal papilla cells were used separately from donated hair follicles. The culture medium was prepared by adding 10% fetal bovine serum (FBS) to high glucose Dulbecco–Vogt modified Eagle's minimal essential medium (DMEM) and placed in a humidified incubator at 37 °C in 5% CO_2_. The culture medium was changed once every 2 ∼ 3 days, and the cells were subcultured several times until a sufficient number of cells was obtained.

Melanocyte differentiation was carried out under specific culture conditions based on the method of Cho et al. [2016]. Melanoblasts were purchased from Creative Bioarray (Shirley, NY) and cultured in Medium 254 (M254; Invitrogen, Waltham, MA) with PMA‐free human melanocyte growth supplement (HMGS‐2; Invitrogen). The cells were placed in a humidified incubator at 37 °C in 5% CO_2_. The culture medium was changed every 2–3 days, and the cells were passaged 3–7 times. To induce differentiation, melanoblasts were exposed to melanocyte differentiation induction medium, consisting of M254, HMGS‐2, 10 nM alpha‐melanocyte stimulating hormone (α‐MSH; M4135, Sigma–Aldrich, St. Louis, MO), 10 nM 12‐*O*‐tetradecanoyl‐phorbol‐13‐acetate (TPA; P1585, Sigma–Aldrich), and 20 μM forskolin (F3917, Sigma–Aldrich). After 3 days, the differentiation induction medium was replaced with M254 before the cells were used.

DPLT spheroids were formed in silicon elastomer‐based concave microwells (StemFIT 3D; MicroFIT, Seongnam, Republic of Korea) with 400 μm diameters. Subsequently, 2.5 × 10^5^ dermal papilla cells were seeded in each concave microwell and cultured with high glucose DMEM + 10% FBS. After dermal papilla cells were seeded for 3 days, 5 × 10^5^ differentiated melanocytes were seeded in each concave microwell containing the dermal papilla spheroids. At this time, the medium was replaced with M254 by removing high glucose DMEM + 10% FBS, and the experiment was conducted 3 days after the melanocytes were seeded.

A total of five DPLT spheroid groups were performed. As controls, a negative control group cultured in M254 and a positive control group cultured in M254 supplemented with 10 mM α‐MSH were used. The experimental groups were divided into three groups: DPLT spheroids treated with RBE (30 μl/ml), EMF, and RBE/EMF.

**Figure 1:**
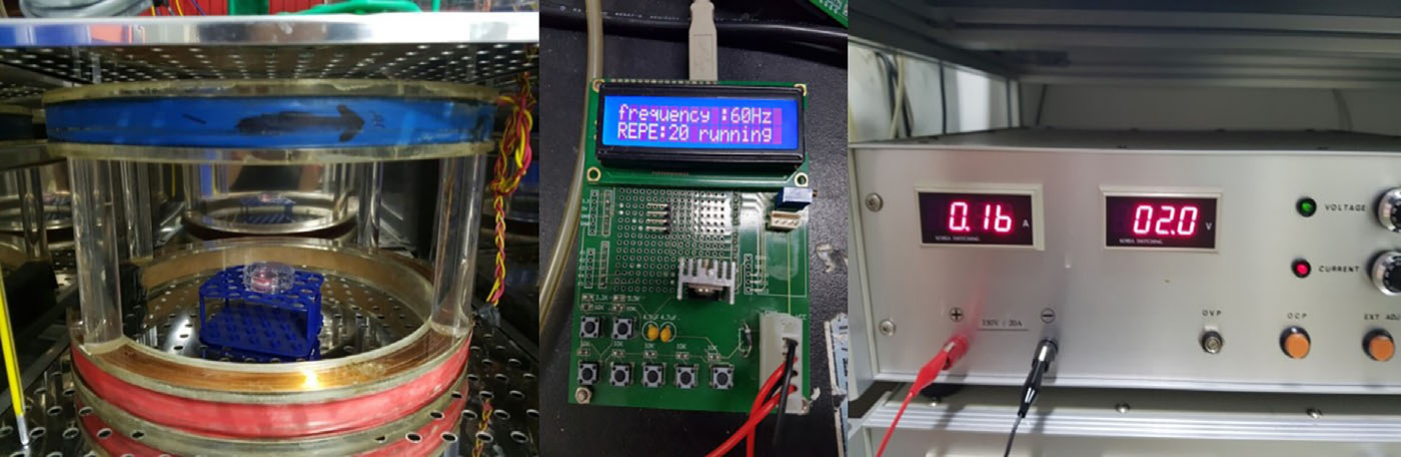
Photograph of the electromagnetic field (EMF) device. Pulsed EMF was generated using a pair of Helmholtz coils of 40 cm diameter and separated by a distance of 15 cm. Coils were placed in a 5% CO_2_ incubator at 37 °C.

The conditions for EMF were based on those used in a study by Cho et al. [2016]. A pair of Helmholtz coils was operated with an alternating current, thereby generating ELF‐EMFs. The ELF‐EMF device was placed in an incubator at 37 °C in 5% CO_2_ and operated at a magnetic field of 0.2 mT, and a frequency of 60 Hz, for 30 min/day for 3 days (Fig. 1).

Cytotoxicity was evaluated by the LDH assay; the medium was collected after cell culture, and an LDH‐LQ kit was used (Asan Pharmaceutical, Seoul, Korea). The medium (100 μl) was placed in a 96‐well plate with 50 μl of working solution and incubated at room temperature for 30 min. Then, the reaction was terminated with 50 μl of stop solution (1 N HCl), and the absorbance was measured at 570 nm.

Tyrosinase activity was measured, based on a method described by Jang and Seo [2016]. The DPLT was treated with RBE, EMF, and RBE/EMF for 3 days. After removing the culture medium from the microwells, the DPLT was washed with phosphate‐buffered saline and lysed with 10% Triton X–100 (93443, Sigma–Aldrich). Then, the DPLT was centrifuged at 15,000 rpm for 15 min, and the protein content of the supernatant was measured by the bicinchoninic acid assay (BCA) (23225, Thermo Fisher Scientific, Waltham, MA). 3,4‐Dihydroxyphenylalanine (L‐DOPA; D9628, Sigma–Aldrich) (10%) was dissolved in sodium phosphate buffer (10 mM). After incubation at 37 °C for 30 min, the absorbance was measured at 475 nm using an ELISA plate reader (PerkinElmer Life Sciences, Waltham, MA).

After culture, the DPLT was lysed, using a buffer containing 2% SDS, 0.1 mg/ml bromophenol blue in Tris‐HCl (pH 6.8), 5% 2‐mercaptoethanol, and 10% glycerol, and boiled at 100 °C for 10 min. The protein was quantified by BCA (23225, Thermo Fisher Scientific). Subsequently, the sample was subjected to SDS‐polyacrylamide gel electrophoresis, and the protein was transferred from the gel to a nitrocellulose membrane. The primary antibodies used were β‐actin (A5441, Sigma–Aldrich), TRP‐1, tyrosinase, MITF, and p‐CREB (ab3312, ab170905, ab12039, and ab32096; Abcam, Cambridge, MA). Blotting images were obtained by the Molecular Imager ChemiDoc XRS+ system with Lumi Femto (Daeil Lab Service, Seoul, Korea). The Western blot images were quantified using ImageJ software (National Institutes of Health, Bethesda, MD).

The overall reverse transcription quantitative real‐time polymerase chain reaction (RT‐qPCR) procedure was based on the experimental method detailed by Cho et al. [2016]. The total RNA of the DPLT was isolated using TRIzol (Invitrogen), according to the standard protocol. RNA concentration was measured by NanoDrop (ND3300LAPTOP, Thermo Fisher Scientific). A total of 2 µg of RNA was used for complementary DNA (cDNA) synthesis, by reverse transcription with an Advantage RT‐PCR kit (Clontech, Palo Alto, CA). The cDNA was subjected to PCR amplification for ET‐1, laminin, bFGF, β‐catenin, MITF, and tyrosinase; the primer sequences are listed in Table 1. Band images were obtained using the Molecular Imager ChemiDoc XRS+ system (Bio‐Rad, Hercules, CA). ImageJ software (National Institutes of Health) was used for the quantitative analysis of RT‐qPCR data.

**Table 1:** Primer Sequence for RT‐PCR

Genes	Upstream primer sequence	Downstream primer sequence
GAPDH	5′‐ACC‐ACA‐GTC‐CAT‐GCC‐ATC‐AC‐3′	5′‐TCC‐ACC‐ACC‐CTG‐TTG‐CTG‐TA‐3′
ET‐1	5′‐GCT‐CGT‐CCC‐TGA‐TGG‐ATA‐AA‐3′	5′‐ATT‐CTC‐ACG‐GTC‐TGT‐TGC‐CT‐3′
Laminin	5′‐TGG‐AGA‐ACG‐CTG‐TGA‐TAG‐GTG‐TCG‐3′	5′‐TGT‐GTA‐AGT‐CTT‐GGT‐GAC‐CCC‐AC‐3′
bFGF	5′‐GGC‐TTC‐TTC‐CTG‐CGC‐ATC‐CAT‐3′	5′‐GGT‐AAC‐GGT‐TAG‐CAC‐ACA‐CTC‐CTT‐T‐3′
β‐catenin	5′‐TGC‐GGA‐CTC‐AGA‐AGG‐AAC‐TCA‐T‐3′	5′‐ACT‐AGT‐CGT‐GGA‐ATG‐GCA‐CC‐3′
MITF	5′‐TTA‐TAG‐TAC‐CTT‐CTC‐TTT‐GCC‐AGT‐CC‐3′	5′‐GTT‐TAT‐TTG‐CTA‐AAG‐TGG‐TAG‐AAA‐GGT‐ACT‐3′
tyrosinase	5′‐CTC‐AAA‐GCA‐GCA‐TGC‐ACA‐AT‐3′	5′‐GCC‐CAG‐ATC‐TTT‐GGA‐TGA‐AA‐3′

Immunohistochemical analysis was conducted based on methods described by Seo et al. [2007] and Kim et al. [2017]. The DPLT was fixed for 2 h at 4 °C with 4% paraformaldehyde. The fixed samples were embedded in paraffin, and 4–5‐μm paraffin sections were stained with hematoxylin and eosin (H&E) and HMB45. Immunohistochemical staining was performed on 4–5‐μm thick serial sections mounted on poly‐L‐lysine‐coated slides. The sections were deparaffinized, and endogenous peroxidase was blocked using 0.03% H_2_O_2_. The nonspecific reaction of bovine serum albumin and non‐immune serum was conducted with the respective monoclonal antibody of anti‐HMB45 (1:1000 dilution; ab787, Abcam) following standard immunohistochemical procedures or with anti‐goat or anti‐rabbit immunoglobulin using the avidin–biotin–peroxidase complex (ABC) method. Either aminoethyl carbazole (AEC) or 3,3′‐diaminobenzidine (DAB) was used as the substrate.

Fontana–Masson silver staining was performed using a previously described method with formalin‐fixed samples stained with silver nitrate (Kojima Chemicals, Kashiwabara, Japan) for 1 h at 56 °C, followed by washing with distilled water. Then, the samples were fixed in 5% sodium thiosulfate solution (S1602, Samchun Pure Chemical, Pyeongtaek, Korea) for 5 min and washed with distilled water. Next, they were stained with nuclear fast red solution (N3020, Sigma–Aldrich) for 5 min and washed three times with distilled water. After dehydration with 95% ethanol and 100% ethanol, the samples were washed two times with xylene (Duksan Pure Chemicals, Seoul, Korea). The F/M and HMB45 staining area densities were normalized using ImageJ software.

### Statistical Analysis

Results are reported as the mean ± standard error (SE), and each experiment was repeated at least three times. Data were analyzed by the Student's *t*‐test. Difference between means were considered significant when *P *< 0.05 (*P < 0.05, **P < 0.01, ***P < 0.005). Graphical representations were produced with SigmaPlot (Systat Software, San Jose, CA).

## RESULTS

**Figure 2:**
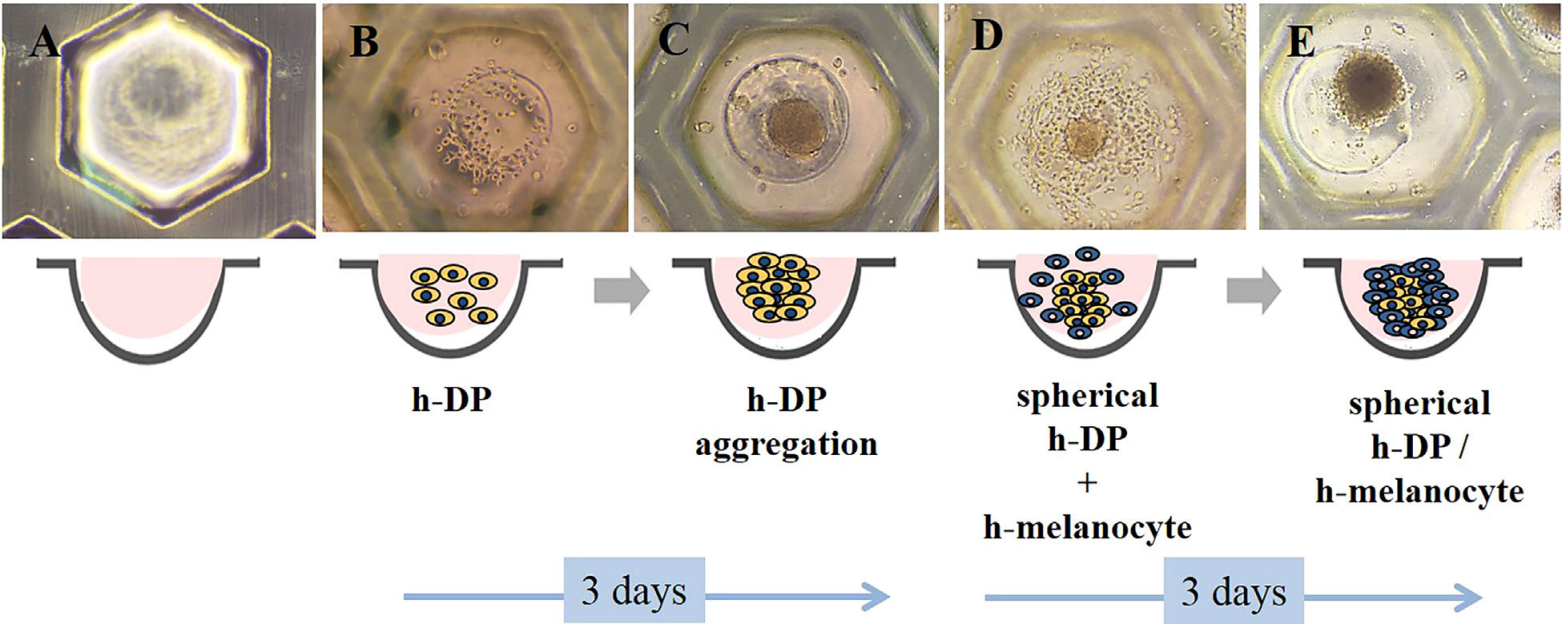
Overall process of forming the DPLT. (A) An image of a microwell without any cells. (B) An image taken immediately after inoculation with dermal papilla cells (DPCs). (C) Sphere formation 3 days after DPC inoculation. (D) Recovered melanocytes that were differentiated with spherical DPCs. (E) DPCs and melanocytes interacted after 3 days to form a spherical shape.

Figure 2 shows the overall process of forming the DPLT. Figure 2A represents one of the 100 microwells without cells. Figure 2B was taken immediately after inoculation with dermal papilla cells. Figure 2C shows the formation of spheres, 3 days after dermal papilla cell inoculation; more than 80% of the cells in the microwells were aggregated and had a diameter of about 100 μm. Some of the dermal papilla cells were spread in wells with poor PHEMA coating. Figure 2D demonstrates the recovery of melanocytes after differentiation and inoculation with aggregated dermal papilla cells, and Figure 2E confirms that after 3 days, the interaction between dermal papilla cells and melanocytes occurred, forming a spherical shape. More than 90% of the melanocytes formed aggregations with dermal papilla cells, and the diameter of the final DPLT was about 130 μm.

**Figure 3:**
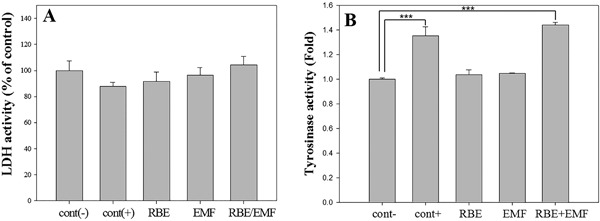
(A) Lactate dehydrogenase (LDH) assay (to examine cellular damage). Each bar represents mean ± standard error (SE) from independent experiments performed in triplicate (*n* = 5). (B) Tyrosinase activity was detected by the tyrosinase activity assay, which was performed after rice bran extract (RBE) and electromagnetic field (EMF) treatment for 3 days. Each bar represents the mean ± SE of independent experiments performed in triplicate (*n* = 3). Significant differences were determined by the Student's *t*‐test; *P < 0.05, **P < 0.01, ***P < 0.005.

The efficacy of RBE and EMF was evaluated using DPLT with a negative control, a positive control, RBE (30 μl/ml), EMF (60 Hz, 0.2 mT, 30 min/day), and RBE/EMF (60 Hz, 0.2 mT, 30 min/day). After 3 days of substance administration and irradiation, the medium in which the cells were cultured was collected to measure the amount of LDH. The LDH assay revealed that the number of cells was similar to or lower than that of the negative control group, and no specific cell damage was observed in the RBE/EMF group (Fig. 3A).

The tyrosinase activity of the DPLT was assayed after RBE addition and EMF irradiation for 3 days. In comparison with the negative control, tyrosinase activity in the experimental group treated with RBE/EMF was found to increase to about 40% (Fig. 3B). However, RBE and EMF did not increase tyrosinase activity in the DPLT. Tyrosinase activity was significantly increased only during simultaneous treatment with RBE/EMF.

**Figure 4:**
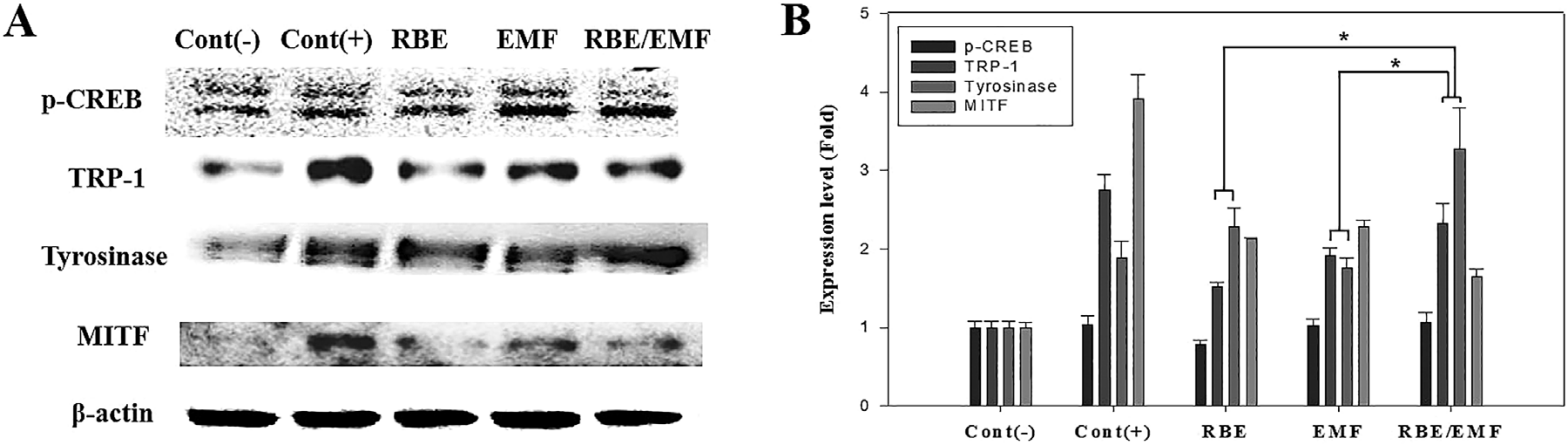
Western blot of protein levels detected in dermal papilla‐like tissue (DPLT) treated with rice bran extract (RBE), electromagnetic field (EMF), and RBE/EMF for 72 h. (A) The protein levels of melanogenesis‐related markers (TRP‐1, tyrosinase, and MITF) and p‐CREB were examined by western blotting. (B) Protein expression of TRP‐1, tyrosinase, MITF, and p‐CREB (with the negative control as the standard). Each bar represents mean ± standard error of independent experiments performed in triplicate (*n* = 3). Significant differences were determined by the Student's *t*‐test; *P < 0.05, **P < 0.01, ***P < 0.005.

As shown in Figure 4A and B, the expression levels of melanogenesis‐related proteins were increased in the RBE, EMF, and EMF/RBE groups compared with the negative control group. Compared to the RBE/EMF‐alone treated experimental groups, the western blot result of the RBE + EMF experimental groups revealed an increase in the expression of TRP‐1 by more than 1.2‐fold and tyrosinase by more than 1.45‐fold.

**Figure 5:**
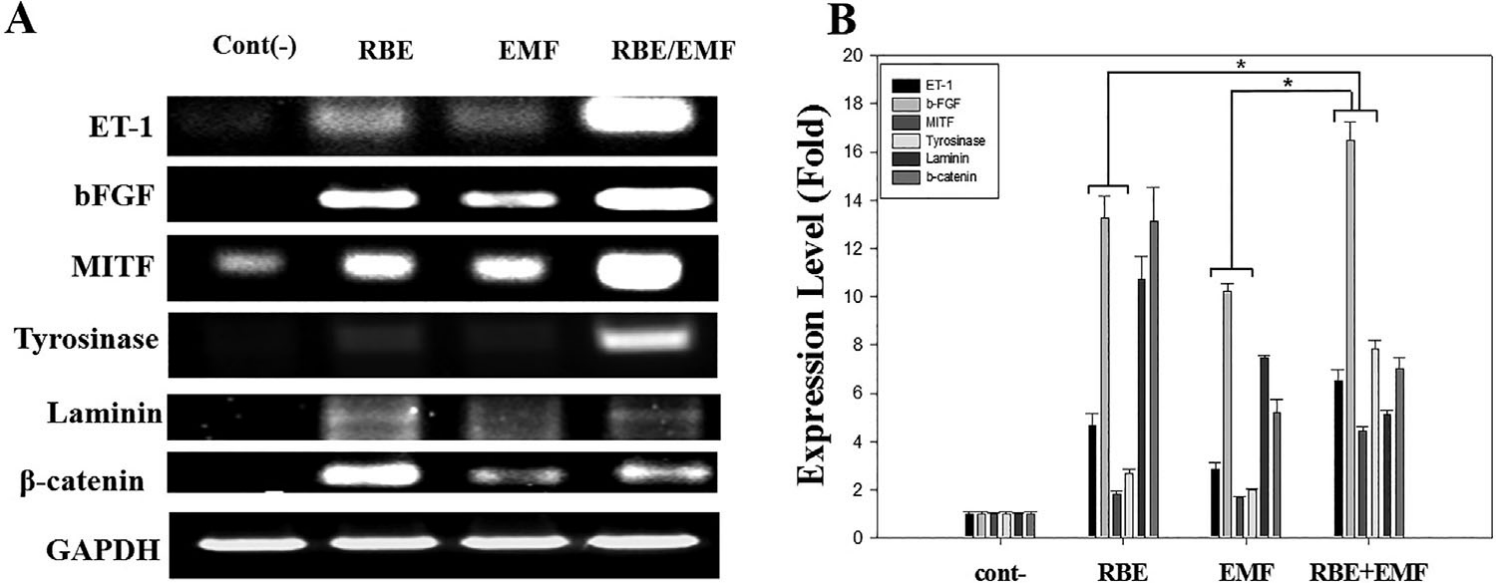
Gene expression detected by reverse transcription polymerase chain reaction (RT‐PCR) on dermal papilla‐like tissue (DPLT) treated with rice bran extract (RBE), electromagnetic field (EMF), and RBE/EMF. (A) mRNA level of ET‐1, laminin, bFGF, β‐catenin, MITF, and tyrosinase. (B) mRNA expression of ET‐1, laminin, bFGF, β‐catenin, MITF, and tyrosinase (with the negative control as the standard). The GAPDH in each lane was used as an internal control. Each bar represents the mean ± standard deviation of independent experiments performed in triplicate. Each bar represents mean ± standard error of independent experiments performed in triplicate (*n* = 3). Significant differences were determined by the Student's *t*‐test; *P < 0.05, **P < 0.01, ***P < 0.005.

In Figure 5A and B, RT‐qPCR analysis revealed markers related to the interactions between dermal papilla cells and melanocytes (ET‐1, laminin, bFGF, and β‐catenin) and melanogenesis‐related markers (MITF and tyrosinase). The mRNA analysis showed that expression levels in the RBE, EMF, and RBE/EMF groups were increased compared with those in the negative control group. Compared to the RBE/EMF‐alone treated experimental groups, the expression levels of ET‐1, b‐FGF, MITF, and Tyrosinase were increased by more than 1.41‐, 1.24‐, 2.58‐, and 2.91‐fold, respectively.

**Figure 6:**
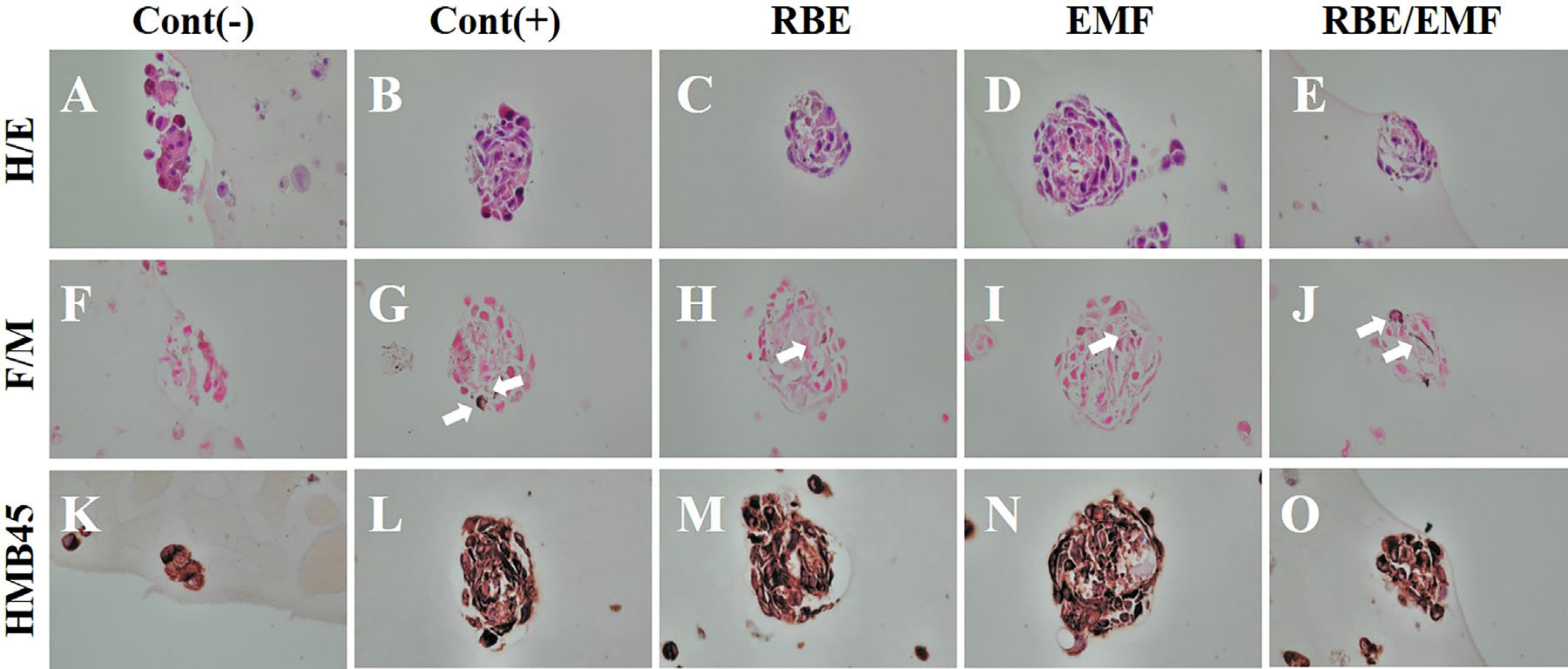
Hematoxylin and eosin (H&E) staining (A–E). Fontana–Masson and HMB45 immunohistochemical staining of the dermal papilla‐like tissue (DPLT) after treatment with rice bran extract (RBE), electromagnetic field (EMF), and RBE/EMF for 3 days. Melanin was visualized by Fontana–Masson silver staining. Melanin is stained dark black (F–J). Light micrograph of human skin melanocytes attached to dish surface, stained with HMB45 for 72 h (K–O).

After RBE addition and EMF irradiation for 3 days, the melanogenesis‐related proteins of the DPLT were evaluated by HMB45 and Fontana–Masson staining. In the experimental groups treated with RBE, EMF, and RBE/EMF, melanin staining was more frequent, and melanin‐producing cells were found. HMB45 staining revealed that melanocytes were stained black and present in the DPLT, in all experimental groups. In the case of Fontana–Masson staining, melanin was increased in the positive control group compared with the negative control group, and melanin was expressed and stained in the RBE/EMF group, as also seen in the positive control group (Fig. 6). Table 2 shows the F/M and HMB45 staining area densities normalized to total volume using ImageJ software.

**Table 2:** The Staining Area Density Was Normalized to Total Volume Using ImageJ Software of F/M and HMB45

	Cont(−)	Cont(+)	RBE	EMF	RBE/EMF
F/M	0.4	1.7	0.5	0.9	4.2
HMB45	9.0	9.2	11.2	8.7	13.7

## DISCUSSION

The effects of exposure to ELF‐EMFs on human health have been widely discussed, with the main concerns focusing on their carcinogenic potential and relationship with immune system functions [Vianale et al., 2008]. However, controlled low‐frequency EMF exposures are still widely used for the treatment of some pathological conditions, to stimulate neural regeneration, as well as tissue and bone repair [Vianale et al., 2008]. Indeed, EMFs have been shown to enhance circulation, by increasing blood flow, and inducing bone formation and healing through increased osteoblast maturation and improved tensile strength recovery after tendon injury [Vianale et al., 2008]. In addition, there have been studies on the promotion of pigmentation using EMFs [Cho et al., 2016].

Various studies have also been conducted on melanogenesis using plant extracts. Kaliziri is a plant that grows only in the south of Xinjiang in China, and the highlands of Pakistan and India. The extract of this plant has been found to increase tyrosinase, MITF, TRP‐1, and TRP‐2 expression [Tuerxuntayi et al., 2014]. Furthermore, Matsuyama et al. [2009] studied *Capparis spinose* plants that were used for spices, diuretics, and hypertension treatment in ancient times and found that the plant extract could increase the melanin content and tyrosinase mRNA of B16 melanoma cells. In another study, Jang and Seo [2016] observed that RBE increased melanin synthesis. Therefore, there have been studies on the synthesis of melanin using RBE [Jang and Seo, 2016] and on increasing melanin using EMF [Cho et al., 2016]. However, since the synergistic effect of RBE and EMF remains unclear, the present study analyzed the melanogenesis activity of RBE and EMF, applied simultaneously.

Based on the tyrosinase analysis in this study, RBE/EMF could be used to promote melanin synthesis in DPLT. In mammals, melanocytes are melanized to produce enzyme‐regulated tyrosinase, TRP‐1, and TRP‐2 [Matsuyama et al., 2009]. Tyrosinase is a catalytic copper‐containing enzyme that converts L‐tyrosine to L‐DOPA and oxidizes L‐DOPA to dopaquinone [Matsuyama et al., 2009; Tuerxuntayi et al., 2014]. TRP‐2, which acts as a DOPA‐chrome tautomerase, catalyzes the rearrangement of DOPA‐chrome to 5,6‐dihydroxyindole‐2‐carboxylic acid (DHICA), and TRP‐1 catalyzes the rearrangement of DHICA to indolequinone. Tyrosinase is the most important enzyme because melanin production depends on tyrosinase expression and activation [Matsuyama et al., 2009; Tuerxuntayi et al., 2014]. In this study, RBE and EMF increased TRP‐1, and in particular, the synergistic effect of RBE/EMF significantly increased tyrosinase.

The tyrosinase family genes (TYR, TRP‐1, and TRP‐2) are tightly regulated by MITF [Tuerxuntayi et al., 2014]. MITF is the most important transcription factor involved in the regulation of TYR gene expression, which is associated with the pigmentation, proliferation, and survival of melanocytes; thus, MITF plays a vital role in melanogenesis [Tuerxuntayi et al., 2014]. The mitogen‐activated protein kinase (MAPK) cascades are an important set of signaling pathways that are activated in response to EMF, as examined in most systems [Wang et al., 2013]. MITF is well known to be controlled by the MAPK signaling pathway [Kim et al., 2017]. In this study, MITF activation was also observed in the EMF and RBE/EMF groups. cAMP stimulates tyrosinase gene expression through activation of cAMP‐dependent protein kinase A (PKA) and CREB transcription factors, thereby increasing the expression of MITF and leading to melanin synthesis [Jung et al., 2011; Amaro‐Ortiz et al., 2014]. p‐CREB can interact with CREB‐binding protein (CBP) to activate MITF, which stimulates tyrosinase gene expression and melanin synthesis [Jiang et al., 2011]. We observed that RBE and EMF activated p‐CREB, and it was significantly increased when simultaneously treated with RBE/EMF. As shown in Figure 4, RBE and EMF increased tyrosinase, TRP‐1, MITF, and p‐CREB of melanocytes in the DPLT.

The dermal papilla cells produce and secrete molecules and growth factors that make up the extracellular matrix (ECM) and cytokines, such as bFGF, ET‐1, and SCF [Lu et al., 2006]. These cytokines migrate to hair matrix cells and induce their differentiation and proliferation [Lu et al., 2006]. The current investigation verified that RBE and EMF increased ET‐1 expression. In particular, the ET‐1 expression in the RBE/EMF group was markedly increased. As intrinsic mediators for human melanocytes, endothelins play vital roles in UVB‐induced pigmentation. Among these endothelin peptides, ET‐1 is considered to be an important member. ET‐1, which was first isolated from vascular endothelial cells, can induce mitogenesis and melanogenesis in primary human melanocytes [Zhang et al., 2013]. Figure 5 shows that ET‐1 levels increased about three‐fold in cells treated with RBE and EMF. More specifically, the expression levels of ET‐1 were increased by more than 17‐fold when simultaneously treated with RBE/EMF. Another study has shown that ET‐1 regulates differentiation and melanogenesis and increases the mRNA level of MC1R [Abdel‐Malek et al., 2000]. Jankovic and Jankovic [1998] reported that bFGF induces stromelysin protein synthesis, promoting the activity and growth of dermal papilla cells. Also, bFGF‐stimulated melanocytes can increase cAMP levels and encourage melanocyte proliferation [Halaban et al., 1987]. From the current investigation, it was evident that RBE and EMF increased bFGF. In particular, bFGF expression was significantly increased in the RBE/EMF group; these results were similar to those of CREB.

Additionally, the ECM component of cultured human dermal papilla cells stimulates the tyrosinase activity of melanocytes [Buffey et al., 1994]. Laminin is expressed in the basement membrane of superficial cells and the ECM of dermal papilla cells in the anagen and catagen phase [Messenger et al., 1991], and it is involved in the attachment of melanocytes [Hara et al., 1994]. Hair pigmentation is associated with the hair cycle, and melanocytes, which are located around the dermal papilla cells, proliferate in the anagen phase of the hair, and initiate melanin synthesis [Buffey et al., 1994; Ideta et al., 2002; Enshell‐Seijffers et al., 2010]. Melanocytes in hair have longer dendrites than melanocytes of the epithelium, and the melanin produced in the mid‐anagen phase is supplied to the end of the hair shaft [Ideta et al., 2002]. During melanin biosynthesis, tyrosinase is synthesized from the initial anagen stage to the late anagen stage and is not synthesized in the catagen and telogen stage [Ideta et al., 2002]. In addition, β‐catenin provides a crucial signal for the telogen‐to‐anagen transition, and the chronic activation of β‐catenin has been found to result in changes consistent with the induction of abnormal growth stages in telogen hair follicles [Krugluger et al., 2011]. Therefore, the anagen phase plays an important role in melanin production in dermal papilla cells. Bellei et al. [2012] noted that β‐catenin has a critical role in prenatal melanocyte biology; they recently demonstrated a physical interaction between CREB and β‐catenin following PKA/cAMP pathway activation in normal human melanocytes and B16‐F0 mouse melanoma cells, which led to the functional cooperation of β‐catenin and CREB on the MITF promoter. The present investigation found that simultaneous stimulation with RBE and EMF did not have a synergistic effect on increasing β‐catenin expression; however, RBE and MITF were able to increase laminin and β‐catenin expression, individually.

We found that the gene expression of dermal papilla cells and melanocytes was increased when DPLT was treated with RBE and EMF. Notably, the overall mRNA and protein expression level in the experimental group treated with both RBE and EMF significantly increased. The increased expression of ET‐1 and bFGF, induced by increasing p‐CREB expression in the group simultaneously administered RBE and EMF, increased melanogenesis.

## CONCLUSION

We demonstrated that the melanocytes of the DPLT were activated by RBE, EMF, and RBE/EMF. RBE, EMF, and RBE/EMF induced the p‐CREB signal, and this signal activated TRP‐1, tyrosinase, and MITF, and increased melanogenesis. Notably, simultaneous stimulation with RBE and EMF had a synergistic effect on increasing melanogenesis. The results of our study can be used as a reference for applying RBE/EMF to a human model. Furthermore, RBE and EMF may be used in the future as a material and therapy, respectively, for the treatment of vitiligo and white hair, through activation of melanogenesis in melanocytes.
